# Chronic Stress and Depression in Periodontitis and Peri-Implantitis: A Narrative Review on Neurobiological, Neurobehavioral and Immune–Microbiome Interplays and Clinical Management Implications

**DOI:** 10.3390/dj10030049

**Published:** 2022-03-18

**Authors:** Francesco D’Ambrosio, Mario Caggiano, Luigi Schiavo, Giulia Savarese, Luna Carpinelli, Alessandra Amato, Alfredo Iandolo

**Affiliations:** Department of Medicine, Surgery and Dentistry “Schola Medica Salernitana”, University of Salerno, Via S. Allende, 84081 Baronissi, Italy; mcaggiano@unisa.it (M.C.); lschiavo@unisa.it (L.S.); gsavarese@unisa.it (G.S.); lcarpinelli@unisa.it (L.C.); aamato@unisa.it (A.A.); aiandolo@unisa.it (A.I.)

**Keywords:** chronic stress, depression, microbiome, periodontitis, peri-implantitis

## Abstract

Besides the well-known systemic factors for periodontal and peri-implant diseases, additional co-factors, such as chronic stress and depression, may also affect disease onset and progression as well as treatment responsiveness. Neurobiological and neurobehavioral pathogenic links between chronic stress and depression, on the one side, and periodontitis and peri-implantitis, on the other side, which have been little investigated and principally related to necrotizing periodontal disease, have been reviewed, along with their putative interconnections with periodontal immune–microbiome balance. Rising evidence suggest that dysregulated neurobiological and neurobehavioral factors, as well as periodontal immune–microbiome unbalance, all related to chronic stress and depression, may crucially interact and thus represent contributing factors in the genesis and worsening not only of necrotizing periodontal lesions, but also of chronic periodontitis and peri-implantitis. Such potential interconnections may be even more relevant in recurrent and aggressive cases of periodontal and peri-implant disease, which are frequently refractory to therapy, and may, if corroborated, coherently pave the way for personalized prevention and treatment strategies, possibly targeting immune–microbiome unbalance and neurobehavioral factors and focusing on neurobiological ones, especially in chronically stressed and depressed subjects with periodontitis and peri-implantitis.

## 1. Introduction

Stress is the psychophysical response to a number of emotional, cognitive or social tasks, perceived by the person as excessive, and comprises a variety of emotional and physiological reactions [1]. The term “stress” was first used by Hans Selye, who defined it as “a non-specific response of the organism to every request made on it”; identified three phases, classified as alarm, resistance and exhaustion phases; and defined it as acute or chronic, based on the duration of the stressful event [2].

Chronic stress has been implied in the onset and development of depression, which is a mood disorder, causing persistent feelings of sadness and loss of interest [3]. Mood disorders, especially the Major Depressive Disorder, are among the most common psychiatric diseases [4]. Major depressive episodes are found both in unipolar disorder, in which mood varies between euthymia and depression, and in bipolar disorder, in which mood has pathological “peaks”, defined as hypomania and mania, as well as euthymia and depression [4]. Subjects with mood disorders also present with altered sleep, appetite and cognition [4,5]; chronic pain; difficulty in carrying out normal daily activities; an increased risk of isolation; and, in the most severe cases, suicidal ideation [6,7]. Indeed, depression is considered one of the leading causes of suicide in the United States, accounting for nearly 50,000 suicides per year [8].

Both chronic stress and depression have been putatively related to periodontitis [9,10]. Periodontitis is an inflammatory disease of bacterial etiology [11], leading to the destruction of the anatomical structures supporting the teeth, bone loss [12,13,14,15], and, eventually, tooth loss. It is one of the main causes of tooth loss in industrialized countries, its prevalence increases with age [16] and it is estimated up to 40% in subjects between 65–74 years of age [17]. Analogously, peri-implantitis is an irreversible inflammatory disease, affecting both soft and hard peri-implant tissue compartments, and it is clinically characterized by peri-implant soft tissue inflammation, with redness, swelling and bleeding on probing, and by peri-implant hard tissues destruction with consequent loss of osseointegration [18,19,20].

Periodontitis and, to a lesser degree, peri-implantitis have been previously associated, in various ways, to a multitude of systemic conditions, such as obesity [21], hyperlipidemia [22] and preterm birth [23]; several inflammatory diseases, including atherosclerosis, rheumatoid arthritis [24], diabetes [25] and inflammatory bowel disease [26]; degenerative disorders such as nervous and macular degenerations [27,28]; and benign and malignant solid neoplasms [29,30]. The proposed link connecting periodontitis and, putatively, peri-implantitis, with such heterogeneous systemic diseases may primarily rely on the common etio-pathogenic factors. In particular, it has been hypothesized that the overall associating mechanisms may result from the interrelation between the underlying systemic inflammation, with a double-way cross-talk between systemic and local periodontal cytokines, a disequilibrium in periodontal microbiome, with dysbiosis, and an altered dynamic balance between host immune response and periodontal microbiome [30,31]. Indeed, although it has been recently proposed that newborns’ oral microbial flora may be strongly influenced by both maternal transfer and genetics, and, once a certain balance between host and periodontal microbiome has been established, any perturbing stimulus is counteracted by restoring the initial genetically determined balance [32], evidence suggests that periodontal microbial flora interacts with the host’s immune system to create a delicate balance. Such periodontal immune–microbiome balance is influenced by genetic and environmental factors, including chronic stress [9]. Accordingly, stress has been previously implied in necrotizing periodontal disease pathogenesis [33,34,35], and such an association has been recently confirmed, considering that during the COVID-19 pandemic, a rising number of necrotizing periodontal lesions have been reported [36,37,38,39,40], along with oral mucosal ones [41], and attributed to the largely increased general stress levels throughout the population if compared to the period before the pandemic [42,43,44,45]. In addition, rising evidence suggests that chronic stress and depression may both affect periodontal and peri-implant tissue homeostasis and thus play a role in periodontal and peri-implant disease onset, severity and response to therapeutic strategies.

Given these considerations, the present literature review aimed, primarily, to evaluate the putative role of stress and depression in periodontitis and peri-implantitis onset, worsening and treatment outcomes, and, secondarily, to point out clinical management implications.

## 2. Methods

Clinical studies and reviews, published in English, with no date restriction, evaluating the potential role of stress and depression in periodontitis and peri-implantitis onset and worsening, were retrieved by two independent reviewers (F.D.A and A.I.) from PubMed and Scopus databases using the following keywords combined by Boolean operators: periodontal OR peri-implant tissue OR health OR disease AND stress AND/OR depression; periodontitis OR peri-implantitis AND stress AND/OR depression. Identified abstracts were independently screened by the two reviewers as well as full-texts independently for pertinent articles; consensus between reviewers was reached through discussion, and eligible articles were considered for the present narrative review.

## 3. Neurobiological Links between Chronic Stress, Depression, Periodontitis and Peri-Implantitis

The adrenergic nerve signaling axis, mainly through norepinephrine, adenosine triphosphate and neuropeptide Y, and the hypothalamic–pituitary–adrenal (HPA) axis, principally through cortisol, physiologically affect the whole body homeostasis along with their pathological activation. These generate a cascade of a wide range of hormones, biologically active peptides and chemokines, which are considered as neurobiological links between chronic stress, depression and several systemic disorders, possibly including periodontitis and peri-implantitis.

### 3.1. Adrenergic Nerve Signaling Axis

The activation of the adrenergic nerve signaling cascade affects the vasculature of postsynaptic smooth muscles, including that in periodontal and peri-implant tissues [9], decreasing blood flow to connective tissue and, consequently, reducing nutrient and cellular diffusion, immune response and wound healing [46,47].

### 3.2. Hypothalamic–Pituitary–Adrenal Axis

The activation of the HPA axis induces the hypothalamus to secrete the corticotropin-releasing hormone and arginine vasopressin, which subsequently promotes the release of adrenocorticotropin from the pituitary gland and successively of glucocorticoids, particularly cortisol, by the adrenal cortex [48]. A chronically dysregulated HPA axis, determining non-physiological cortisol levels, as well as adrenal disorders have been related to both chronic stress [49,50] and depression [51,52]. Consequently, considering the well-known effects glucocorticoids, responsible for maintaining body homeostasis, on the HPA, depression and chronic stress may, altering cortisol levels, negatively affect blood pressure, sodium retention, intra-abdominal visceral fat accumulation, insulin resistance, and, notably, inflammatory cytokine secretion [49,50,53,54,55,56]. In addition, it has been reported that keratinocytes from periodontal and peri-implant tissues physiologically express glucocorticoid receptors and both respond to the HPA cortisol and secrete cortisol themselves as an autocrine factor for soft tissue homeostasis [57]. It has also been demonstrated that excess cortisol levels may delay periodontal wound healing and be associated with periodontitis worsening [58] by means of cytokines modulation [49].

#### Hypothalamic–Pituitary–Adrenal Axis and Periodontal Pro-Inflammatory Cytokines

Cytokines are signaling proteins that have the main function of transmitting information between the central nervous system, endocrine system and immune cells [59]. In the chronic inflammatory state associated with stress, Interleukin (IL)-1beta, Tumor Necrosis Factor (TNF)-alpha and IL-6 have been reported to be the most represented cytokines [49]; Serum C-Reactive Protein, IL-1 and IL-6 were the most prevalent in depression [60,61].

Concordantly, elevated levels of IL-1beta were found in patients who presented psychological stress and periodontitis [62,63,64], and increased levels of cytokines reducing host response were associated with periodontitis worsening [65].

In addition, chronic stress was also related to delayed wound healing in periodontal and peri-implant soft tissues, secondary to reduced levels of IL-1alfa and beta, IL-6, IL-8 and TNF-alfa [66], as well as hard tissues, thus favoring periodontitis progression and poor osseointegration [9,49].

## 4. Neurobehavioral Links between Chronic Stress, Depression, Periodontitis and Peri-Implantitis

### 4.1. Health-Related Behaviors

Physically adaptive changes in hippocampal morphology may determine behavior modifications related to hygiene practice, lethargy, lack of organization, anxiety and compliance to treatment and may, consequently, represent the neurobehavioral links between chronic stress, depression, periodontitis and peri-implantitis [9]. Indeed, chronic stress and depression may initiate, through epigenetic modifications, such physically adaptive changes in different hippocampal subregions [67,68]. Accordingly, changes in health-related behaviors due to chronic stress and depression, and causing poor oral hygiene and adherence to both active and maintenance phases of periodontal treatment, have been implied in periodontitis onset [10] and progression [69] and in increased tooth loss [70].

### 4.2. Health-Damaging Behaviors

Moreover, chronic stress and depression, besides those changes in health-related behaviors, may also lead to the assumption of health-damaging behaviors, such as tobacco smoking, which is a well-recognized risk factor for periodontitis [71]; alcohol consumption, implicated in tooth loss [72]; and unhealthy diets, promoting dysbiosis [73] and favoring an inflammatory environment, common to those comorbidities implied in periodontitis grading and progression rate and to those systemic disorders linked to periodontal disease [74].

## 5. Periodontal Immune–Microbiome Balance in the Link between Chronic Stress, Depression, Periodontitis and Peri-Implantitis

It has been proposed that chronic stress may alter the composition of the commensal microbiota in the human microbiome [75], resulting in the so-called stress-related dysbiosis. It is well known that a dysbiotic microbiome may lead to a series of different diseases [48,76,77]. Periodontitis and peri-implantitis, which are initiated by bacterial aggregation, leading to periodontal and peri-implant tissues inflammation, subsequently progressing in an apical direction and invading bone compartment [11,19], are also characterized by a dysbiotic microbiota, with decreased coccoid and straight rod microbial populations and increased motile organisms compared to periodontally healthy sites [78].

### 5.1. Stress-Related Periodontal Dysbiosis

It has been hypothesized that oral and periodontal microorganisms may have evolved evolutionary systems, primarily for sensing host-associated signals altering their environment as hormones, and secondarily for properly adapting their gene expression profile to the new environmental conditions [48].

In addition, it has been reported that higher cortisol levels were detected in gingival crevicular fluid from subjects suffering from periodontitis [79], and that induced a shift in periodontal microbiome profile expression typically observed in periodontitis progression [80,81]. In particular, periodontal Fusobacteria species significantly increased the number of transcripts after the experimental addition of cortisol [80,81]. From this perspective, the periodontal microbiome may be capable of sensing changes in stress hormone levels and may consequently react, affecting periodontal and peri-implant health conditions and diseases.

### 5.2. Stress-Related Periodontal Immune Deficiency

It has been demonstrated that prolongated high cortisol levels may reduce immune cell activity, modifying the T-helper and T-suppressor lymphocyte balance and altering Natural Killer cells functioning [82,83]. Consequently, chronic stress and depression may indirectly promote microbial infection onset and worsening and may increase pro-inflammatory cytokines, causing, in turn, a mild chronic inflammation [48].

## 6. Stress, Depression, Periodontitis, Peri-Implantitis and COVID-19: The Role of Cytokines

A plethora of symptoms and signs, potentially related to both severe acute respiratory syndrome coronavirus 2 (SARS-CoV-2) infection and to the Coronavirus Disease 2019 (COVID-19) illness itself, and also involving the oral cavity, have been described [41]. In addition to the well-known clinical presentation of COVID-19, several studies have also reported psychiatric sequelae that may be secondary to both the immune response to the virus itself and to psychological stressors [84]. Indeed, the immune response to SARS-CoV-2 induces a “cytokine storm” that consists in an uncontrolled and dysregulated local and systemic production of cytokines, chemokines and other inflammatory mediators, especially IL-1beta, IL-6 and Interferon (IFN)-γ, suggesting the activation of T-helper-1 lymphocytes, as well as IL-4 and IL-10, secreted, instead, by T-helper-2 lymphocytes [85].

A similar dysregulation of cytokines is also described in stress and depression as well as in periodontal and peri-implant diseases [30,31,60,86]. Indeed, pro-inflammatory cytokines were found to be upregulated and, conversely, anti-inflammatory cytokines were downregulated through different mechanisms, involving the HPA “fatigue”, the resistance to glucocorticoids, the activation of inflammation-related transcription pathways and the negative feedback from the body in chronic stress [86]. A recent metanalysis reported an increase in systemic pro-inflammatory cytokines, IL-1, IL-6, TNF-alpha and C-reactive protein (CRP) in depression patients [60]. Furthermore, a dysregulation of systemic pro-inflammatory cytokines, such as IL-1beta, IL-6, IL-12, TNF-alfa, regulatory cytokines including IL-4, IL-1RA, IL-10 and interferon (IFN)-γ, is very important also in the pathogenesis and in the worsening of periodontal and peri-implant disease [30,31]. A similar COVID-19 cytokine dysregulation, in particular of IL-1beta, IL-6, IL-10, IFN-γ, TNF-alfa and Transforming Growth Factor (TGF)-beta, was found in patients who presented psychiatric disorders, periodontitis and peri-implant disease [9].

## 7. Clinical Management Implications

Based on individual predisposing conditions, stress and depression may heterogeneously affect periodontitis and peri-implant disease onset and severity due to the individual innate capacity to maintain a homeostatic balance in circulating hormones and chemokines [9].

Moreover, the inflammatory response and the consequent alteration of cytokine levels has been found similar in stress and depression, on the one side, and in periodontal and peri-implant diseases, on the other side [9], highlighting the possibility as well as the necessity of targeting shared pathogenic mechanisms in order to comprehensively manage such complex multi-factorial disorders and, especially, periodontitis and peri-implantitis. In particular, implementing the whole series of measures and interventions aiming to eliminate or, at least, to control risk factors, pathogenic mechanisms and clinical signs related to periodontitis and peri-implantitis might be of crucial importance both for the population-based and high-risk surveillance and for integrated therapeutic approaches to periodontal and peri-implant diseases.

### 7.1. Periodontitis and Peri-Implantitis Prevention

Primary and secondary prevention strategies may be considered even more relevant in subjects suffering from depression or chronic stress in order to comprehensively minimize the risk of periodontal and peri-implant disease onset and progression and to reduce the development of several oral disorders, mainly related to poor self-care and deficient immune response, as illustrated above, as well as of systemic diseases, principally gastrointestinal ones [87], thus positively affecting oral and general health.

An improvement in health-related behaviors should be encouraged through education and reinforcements, both during operative sessions and through teledentistry tools and apps [37]. Specifically, stressed and depressed patients should be kept even more motivated, compared to periodontal subjects not suffering from chronic stress and depression, to maintain good oral hygiene and to adhere to active and maintenance phases [10,69].

Moreover, the elimination of frequently acquired health-damaging behaviors should also be targeted, especially in stressed and depressed periodontal patients, focusing on smoking habits, alcohol consumption, poor sleep quality and nutrition [71,72,73,74]. In particular, when harmful behaviors emerge, it may be appropriate to refer patients to specialists helping them to quit smoking and alcohol abuse and sleep better, and to give them nutritional advice [9]. Out of all the prevention measures controlling health-damaging behaviors, smoking cessation certainly remains one of the most favorable prognostic factors for maintaining good periodontal and peri-implant health [9].

Periodontitis and peri-implantitis prevention in stressed and depressed subjects should also aim to manage those factors that contribute to aggravating anxiety, depression and stress; indeed, trying to mitigate these pathological states has been proven to positively affect general health as well as oral health [9]. In more detail, a recent study investigated the effectiveness of yoga in association with periodontitis and peri-implantitis therapies and of meditation practices in the management of periodontal and peri-implant diseases and evaluated stress influence on treatment outcomes [88]. The results highlighted that periodontal and peri-implant treatment combined with yoga was associated with a reduction in the stress levels perceived by the subjects and with an improvement in periodontal and peri-implant health, shown by a higher reduction in probing depth values when compared to standard treatment for periodontal and peri-implant diseases [88].

### 7.2. Periodontitis and Peri-Implantitis Therapy

Scheduling appointments early in the morning, in order to minimize the stress and anxiety of waiting that could build up during the day, could help the clinician in the treatment of patients with stress and depression; similarly, in order to reduce the patient’s anxiety, simple and conservative treatment techniques should be preferred, also minimizing the risk of post-operative complications [9]. Moreover, short operative sessions should be favored as they may make stressed and depressed patients feel more comfortable and have been reported to be sufficient to maintain good periodontal and peri-implant health [9]. Furthermore, short periodontal and peri-implant maintenance reminders may aid in preserving both compliance and adherence to treatment of chronically stressed and depressed subjects [9]; in fact, since those patients are very often unmotivated, it is very important to emphasize the importance of not to forget nor to skip appointments. Additionally, the use of oral antimicrobials, such as 0.2% chlorhexidine mouthwash, may aid in the control of the overall periodontal and peri-implant microbial load between sessions and in soft tissue healing following surgery [9]. Some authors have also proposed the administration of antibiotics as adjuncts to mechanical periodontal and peri-implant treatments in order to improve treatment outcomes. In particular, the administration of low-dose doxycycline could also be recommended as an adjunct in stressed and depressed patients since it has been demonstrated to effectively support both supragingival and subgingival debridement and improve clinical periodontal and peri-implant indices, particularly probing depth and bleeding on probing. Antibiotic prophylaxis may even be considered in subjects suffering from chronic stress and depression with reduced immune competence from the perspective of preventing complications.

Particular attention should also be given to pain management during periodontal and peri-implant procedures in subjects with mood disorders. Indeed, an increase in perceived post-operative pain, related to an increased use of analgesic drugs, has been reported both in subjects who presented higher levels of pre-operative stress or anxiety and in those suffering from depression [89]. As a counterpart, patients taking antidepressants reported less susceptibility to post-operative pain and used a lower number of analgesics compared to those not taking antidepressants [89]. However, patients undergoing periodontal surgery who were treated with interventions to minimize post-operative anxiety and stress showed less post-operative pain compared to those who did not receive the same interventions to minimize stress [89], strengthening the hypothesis of a close relation between stress, depression and pain, where stress and depression might be considered as predictors of post-operative pain [90].

A strong relation has also been hypothesized between stress and depression on the one side and periodontal and peri-implant tissue healing and disease severity, as discussed above, on the other side [9,49,91]. Concordantly, stressed subjects who underwent pre-operative relaxation techniques showed better healing of periodontal wounds, following surgical procedures, compared to patients who did not undergo relaxation treatments [90]. Moreover, Kiecolt-Glaser et al. highlighted how stress negatively affects the cellular immune response, resulting in a delay in wound healing in depressed patients compared to non-depressed patients [91]. Takada et al. showed that increasing stress levels result in a faster progression of periodontal and peri-implant disease. This is probably determined by a greater production of IL-1, IL-4 and IL-8, which are involved in the inflammatory process of periodontal and peri-implant destruction [92].

### 7.3. Psychological Interventions for Periodontal and Peri-Implant Health

Oral and periodontal health information and education, aiming to increase patients’ periodontal awareness and literacy, are mainly focused on the improvement in health-related behaviors and on the elimination of health-damaging behaviors, therefore constituting the key points for effective preventive and therapeutic periodontal and peri-implant strategies [93]. However, periodontal education has been proved to lead to poor and only short-term effects on oral health improvement and to limitedly affect the overall periodontal outcomes [94], advocating the need for additional multi-disciplinary interventions in the integrated management of periodontal and peri-implant diseases.

Since psychological factors, such as oral health beliefs and dental anxiety, affect oral-health-related behaviors, mainly including oral hygiene habits, diet, smoking and compliance to periodontal and peri-implant therapy, we believe that psychological interventions may guide behavioral changes and may, as a consequence, positively affect periodontitis and peri-implantitis prevention and treatment outcomes, although inconclusive results have been previously reported [94]. Conversely, plaque control was slightly improved, subsequent to psychological intervention, when compared to periodontal health education [93], potentially highlighting the role of such an additional approach in periodontal reinforcement. Therefore, considering time and cost efforts, we believe that integrating psychological intervention in periodontal and peri-implant health management may be crucial, especially in refractory and recurrent cases. From this perspective, interdisciplinary collaboration should be taken into account and clinicians should be properly educated to assess both specific patient behaviors towards periodontal and peri-implant health and ability as well as motivation to modify such behaviors [95] to effectively communicate and motivate the maintenance of good oral hygiene and encourage compliance to treatment [96], actively involving the patient in the overall periodontal and peri-implant care.

## 8. Conclusions

Although available data are still very few, compounding evidence suggests that, besides individual, genetic and systemic factors [32,97,98,99], also additional co-factors, comprising chronic stress and depression, may play a role in periodontal and peri-implant disease onset and progression, and, consequently, affect treatment responsiveness. 

Neurobiological and neurobehavioral pathogenic links between chronic stress, depression and systemic disorders have been described, besides the fact that their role in periodontal and peri-implant diseases has been little investigated, and principally related to necrotizing periodontal disease.

**Figure 1:**
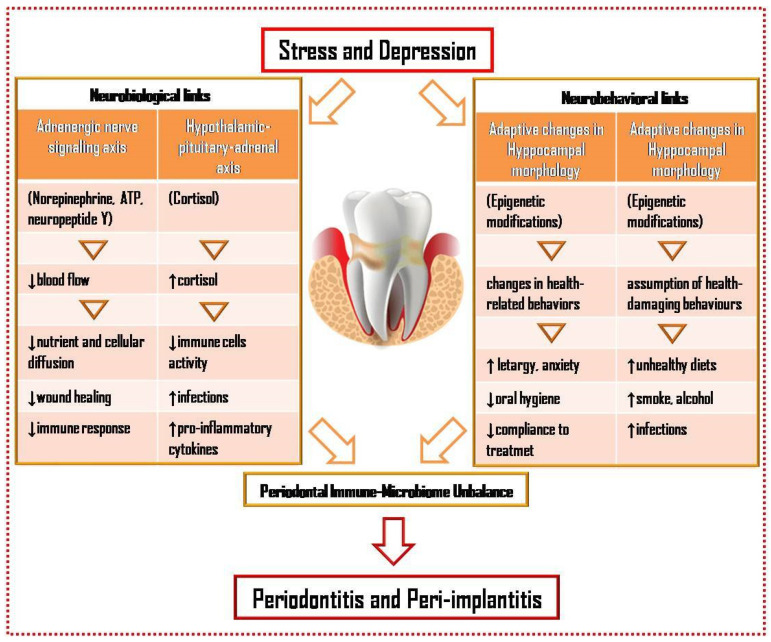
Neurobiological and neurobehavioral links and periodontal immune–microbiome unbalance in the possible association between chronic stress, depression and periodontal and peri-implant diseases.

However, it could be speculated that, under chronic stress, periodontal and peri-implant tissues may be characterized by a pro-inflammatory and anti-regenerative milieu, with increased infection susceptibility and decreased healing capacity, and by a reduced immune response, that may, all together, cause periodontal microbiome dysbiosis (Figure 1). As a counterpart, dysbiosis itself may, along with the infectious–inflammatory processes generated, trigger stress and alter the immune response [9].

Such emerging compound evidence may pave the way for a more comprehensive management of periodontal and peri-implant tissue health management and for more integrated periodontitis and peri-implantitis prevention and treatment plans in chronically stressed and depressed subjects and especially targeting specific risk factors and pathogenic mechanisms.

### Future Directions

Given these considerations, it could be speculated that dysregulated neurobiological and neurobehavioral factors, as well as periodontal immune–microbiome unbalance, all related to chronic stress and depression, may crucially interplay and thus represent contributing factors not only in the genesis and worsening of necrotizing periodontal lesions, but also of chronic periodontitis and peri-implantitis. Further studies are needed, corroborating these findings, especially considering that such potential interconnections may be even more relevant in recurrent and aggressive cases of periodontal and peri-implant disease, which are frequently refractory to therapy, and may, coherently, pave the way for personalized prevention and treatment strategies, potentially targeting immune–microbiome unbalance and neurobehavioral factors and focusing on neurobiological ones, especially in chronically stressed and depressed subjects with periodontitis and peri-implantitis.

## Data Availability

PubMed and Scopus databases.

## References

[1] Brown S.M., Doom J.R., Lechuga-Pena S., Watamura S.E., Koppels T. (2020). Stress and parenting during the global COVID-19 pandemic. Child Abuse Negl..

[2] Selye H. (1950). Stress and the general adaptation syndrome. Br. Med. J..

[3] Tafet G.E., Bernardini R. (2003). Psychoneuroendocrinological links between chronic stress and depression. Prog. Neuropsychopharmacol. Biol. Psychiatry.

[4] Beurel E., Toups M., Nemeroff C. (2020). The Bidirectional Relationship of Depression and Inflammation: Double Trouble. Neuron.

[5] American Psychiatric Association (2013). Diagnostic and statistical manual of mental disorders. BMC Med..

[6] Ruggles K.V., Fang Y., Tate J., Mentor S.M., Bryant K.J., Fiellin D.A., Justice A.C., Braithwaite R.S. (2017). What are the Patterns between Depression, Smoking, Unhealthy Alcohol Use, and Other Substance Use among Individuals Receiving Medical Care? A Longitudinal Study of 5479 Participants. AIDS Behav..

[7] Ribeiro J.D., Huang X., Fox K.R., Franklin J.C. (2018). Depression and hopelessness as risk factors for suicide ideation, attempts and death: Meta-analysis of longitudinal studies. Br. J. Psychiatry.

[8] Brody D.J., Pratt L.A., Hughes J.P. (2018). Prevalence of depression among adults aged 20 and over: United States, 2013–2016. NCHS Data Brief.

[9] Decker A.M. (2021). The psychobiological links between chronic stress-related diseases, periodontal/peri-implant diseases, and wound healing. Periodontol. 2000.

[10] Dumitrescu A.L. (2016). Depression and Inflammatory Periodontitis Considerations. Front. Psychol..

[11] Tonetti M.S., Greenwell H., Kornman K.S. (2018). Staging and grading of periodontitis: Framework and proposal of a new classification and case definition. J. Periodontol..

[12] Ramaglia L., Di Spirito F., Sirignano M., La Rocca M., Esposito U., Sbordone L. (2019). A 5-year longitudinal cohort study on crown to implant ratio effect on marginal bone level in single implants. Clin. Implant Dent. Relat. Res..

[13] Guevara Perez S.V., De la Rosa Castolo G., Thollon L., Behr M. (2018). A 3D characterization method of geometric variation in edentulous mandibles. Morphologie.

[14] Sbordone C., Toti P., Brevi B., Martuscelli R., Sbordone L., Di Spirito F. (2018). Computed tomography-aided descriptive analysis of maxillary and mandibular atrophies. J. Stomatol. Oral Maxillofac. Surg..

[15] Di Spirito F., Toti P., Brevi B., Martuscelli R., Sbordone L., Sbordone C. (2019). Computed tomography evaluation of jaw atrophies before and after surgical bone augmentation. Int. J. Clin. Dent..

[16] Papapanou P.N., Susin C. (2017). Periodontitis epidemiology: Is periodontitis underrecognized, over-diagnosed, or both?. Periodontol. 2000.

[17] World Health Organization World Oral Health Report 2018/2019.

[18] Renvert S., Persson G.R., Pirih F.Q., Camargo P.M. (2018). Peri-implant health, peri-implant mucositis, and peri-implantitis: Case definitions and diagnostic considerations. J. Clin. Periodontol..

[19] Berglundh T., Armitage G., Araujo M.G., Avila-Ortiz G., Blanco J., Camargo P.M., Chen S., Cochran D., Derks J., Figuero E. (2018). Peri-implant diseases and conditions: Consensus report of workgroup 4 of the 2017 World Workshop on the Classification of Periodontal and Peri-Implant Diseases and Conditions. J. Clin. Periodontol..

[20] Araujo M.G., Lindhe J. (2018). Peri-implant health. J. Clin. Periodontol..

[21] Di Spirito F., Sbordone L., Pilone V., D’Ambrosio F. (2019). Obesity and Periodontal Disease: A Narrative Review on Current Evidence and Putative Molecular Links. Open Dent. J..

[22] Di Spirito F., Schiavo L., Pilone V., Lanza A., Sbordone L., D’Ambrosio F. (2021). Periodontal and peri-implant diseases and systemically administered statins: A systematic review. Dent. J..

[23] Sert T., Kırzıoğlu F.Y., Fentoğlu O., Aylak F., Mungan T. (2011). Serum placental growth factor, vascular endothelial growth factor, soluble vascular endothelial growth factor receptor-1 and -2 levels in periodontal disease, and adverse pregnancy outcomes. J. Periodontol..

[24] Soory M. (2010). Association of periodontitis with rheumatoid arthritis and atherosclerosis: Novel paradigms in etiopathogeneses and management?. Open Access Rheumatol..

[25] Chapple I.L.C., Genco R., on behalf of working group 2 of the joint EFP/AAP workshop (2013). Diabetes and periodontal diseases: Consensus report of the Joint EFP/AAPWorkshop on Periodontitis and Systemic Diseases. J. Periodontol..

[26] Lauritano D., Sbordone L., Nardone M., Iapichino A., Scapoli L., Carinci F. (2017). Focus on periodontal disease and colorectal carcinoma. Oral Implantol..

[27] Elemek E., Almas K. (2013). Multiple sclerosis and oral health: An update. N. Y. State Dent. J..

[28] Di Spirito F., La Rocca M., De Bernardo M., Rosa N., Sbordone C., Sbordone L. (2020). Possible Association of Periodontal Disease and Macular Degeneration: A Case-Control Study. Dent. J..

[29] Michaud D.S., Liu Y., Meyer M., Giovannucci E., Joshipura K. (2008). Periodontal disease, tooth loss, and cancer risk in male health professionals: A prospective cohort study. Lancet Oncol..

[30] Di Spirito F., Toti P., Pilone V., Carinci F., Lauritano D., Sbordone L. (2020). The Association between Periodontitis and Human Colorectal Cancer: Genetic and Pathogenic Linkage. Life.

[31] Lee J.Y., Park H.J., Lee H.J., Cho H.J. (2019). The use of an interdental brush mitigates periodontal health inequalities: The Korean National Health and nutrition examination survey (KNHANES). BMC Oral Health.

[32] Curtis M.A., Diaz P.I., Van Dyke T.E. (2020). The role of the microbiota in periodontal disease. Periodontol. 2000.

[33] Herrera D., Retamal-Valdes B., Alonso B., Feres M. (2018). Acute periodontal lesions (periodontal abscesses and necrotizing periodontal diseases) and endo-periodontal lesions. J. Clin. Periodontol..

[34] Coppola C., Mollo M., Pacelli T. (2019). The worlds’ game: Collective language manipulation as a space to develop logical abilities in a primary school classroom. Eur. J. Psychol. Educ..

[35] Savarese G., Fasano O., Mollo M., Pecoraro N. (2013). From Personal Identity to Pluralism of Intercultural Identity: A study on the transferability of self-knowledge to the multicultural social contexts. Knowl. Cult..

[36] Aragoneses J., Suarez A., Algar J., Rodriguez C., Lopez-Valverde N., Aragoneses J.M. (2021). Oral Manifestations of COVID-19: Updated Systematic Review with Meta-Analysis. Front. Med..

[37] Di Spirito F., Iacono V.J., Iandolo A., Amato A., Sbordone L., Lanza A. (2021). Evidence-based recommendations on periodontal practice and the management of periodontal patients during and after the COVID-19 era: Challenging infectious diseases spread by airborne transmission. Open Dent. J..

[38] Iannelli A., Favre G., Frey S., Esnault V., Gugenheim J., Bouam S., Schiavo L., Tran A., Alifano M. (2020). Obesity and COVID-19: ACE 2, the Missing Tile. Obes. Surg..

[39] Martina S., Amato A., Rongo R., Caggiano M., Amato M. (2020). The Perception of COVID-19 among Italian Dentists: An Orthodontic Point of View. Int. J. Environ. Res. Public Health.

[40] Ammar N., Aly N.M., Folayan M.O., Khader Y., Virtanen J.I., Al-Batayneh O.B., Mohebbi S.Z., Attia S., Howaldt H.P., Boettger S. (2020). Behavior change due to COVID-19 among dental academics—The theory of planned behavior: Stresses, worries, training, and pandemic severity. PLoS ONE.

[41] Di Spirito F., Pelella S., Argentino S., Sisalli L., Sbordone L. (2020). Oral manifestations and the role of the oral healthcare workers in COVID-19. Oral Dis..

[42] Kannampallil T.G., Goss C.W., Evanoff B.A., Strickland J.R., McAlister R.P., Duncan J. (2020). Exposure to COVID-19 patients in- creases physician trainee stress and burnout. PLoS ONE.

[43] Barzilay R., Moore M.T., Greenberg D.M., DiDomenico G.E., Brown L.A., White L.K., Gur R.C. (2020). Resilience, COVID-19-related stress, anxiety and depression during the pandemic in a large population enriched for healthcare providers. J. Transl. Psychiatry.

[44] Manea A., Crisan D., Baciut G., Baciut M., Bran S., Armencea G., Crisan M., Colosi H., Colosi I., Vodnar D. (2021). The importance of atmospheric microbial contamination control in dental offices: Raised awareness caused by the SARS-CoV-2 pandemic. Appl. Sci..

[45] Savarese G., Iannaccone A., Mollo M., Pecoraro N., Fasano O., Carpinelli L. (2019). Academic Performance-related Stress Levels and Reflective Awareness: The Role of the Elicitation Approaching an Italian University’s Psychological Counselling. Br. J. Guid. Couns..

[46] Prete A., Taylor A.E., Bancos I., Smith D.J., Foster M.A., Kohler S., Fazal-Sanderson V., Komninos J., O’Neil D.M., Vassiliadi D.A. (2020). Prevention of adrenal crisis: Cortisol responses to major stress compared to stress dose hydrocortisone delivery. J. Clin. Endocrinol. Metab..

[47] Felten D.L., Felten S.Y., Carlson S.L., Olschowka J.A., Livnat S. (1985). Noradrenergic and peptidergic innervation of lymphoid tissue. J. Immunol..

[48] Duran-Pinedo A.E., Solbiati J., Frias-Lopez J. (2018). The effect of the stress hormone cortisol on the metatranscriptome of the oral microbiome. NPJ Biofilms Microbiomes.

[49] Warren K.R., Postolache T.T., Groer M.E., Pinjari O., Kelly D.L., Reynolds M.A. (2014). Role of chronic stress and depression in periodontal diseases. Periodontol. 2000.

[50] Breivik T., Thrane P.S., Murison R., Gjermo P. (1996). Emotional stress effects on immunity, gingivitis and periodontitis. Eur. J. Oral Sci..

[51] Miller A.H., Maletic V., Raison C.L. (2009). Inflammation and its discontents: The role of cytokines in the pathophysiology of major depression. Biol. Psychiatry.

[52] Belvederi Murri M., Pariante C., Mondelli V., Masotti M., Atti A.R., Mellacqua Z., Antonioli M., Ghio L., Menchetti M., Zanetidou S. (2014). HPA axis and aging in depression: Systematic review and meta-analysis. Psychoneuroendocrinology.

[53] Bautista L., Bautista L.E., Bajwa P.K., Shafer M.M., Malecki K.M.C., McWilliams C.A., Palloni A. (2019). The relationship between chronic stress, hair cortisol and hypertension. Int. J. Cardiol. Hypertens..

[54] Mocayar Marón F.J., Ferder L., Daniel Saravì F., Manucha W. (2019). Hypertension linked to allostatic load: From psychosocial stress to inflammation and mitochondrial dysfunction. Stress.

[55] Quax R.A., Manenscijn L., Koper J.W., Hazes J.M., Lamberts S.W.J., van Rossum E.F.C., Feelders R.A. (2013). Glucocorticoid sensitivity in health and disease. Nat. Rev. Endocrinol..

[56] Cohen S., Janicki-Deverts D., Doyle W.J., Miller G.E., Frank E., Rabin B.S., Turner R.B. (2012). Chronic stress, glucocorticoid receptor resistance, inflammation, and disease risk. Proc. Natl. Acad. Sci. USA.

[57] Cirillo N., Prime S.S. (2011). Keratinocytes synthesize and activate cortisol. J. Cell. Biochem..

[58] Ishisaka A., Ansai T., Soh I. (2008). Association of cortisol and dehydroepiandrosterone sulphate levels in serum with periodontal status in older Japanese adults. J. Clin. Periodontol..

[59] Cannon J.G. (2000). Inflammatory cytokines in nonpathological states. News Physiol. Sci..

[60] Howren M.B., Lamkin D.M., Suls J. (2009). Associations of depression with C-reactive protein, IL-1, and IL-6: A meta-analysis. Psychosom. Med..

[61] Hiles S.A., Baker A.L., de Malmanche T., Attia J. (2012). A meta-analysis of differences in IL-6 and IL-10 between people with and without depression: Exploring the causes of heterogeneity. Brain Behav. Immun..

[62] Deinzer R., Kottmann W., Förster P., Herforth A., Stiller-Winkler R., Idel H. (2000). After-effects of stress on crevicular interleukin-1beta. J. Clin. Periodontol..

[63] Giannopoulou C., Kamma J.J., Mombelli A. (2003). Effect of inflammation, smoking and stress on gingival crevicular fluid cytokine level. J. Clin. Periodontol..

[64] Lopez-Castejon G., Brough D. (2011). Understanding the mechanism of IL-1beta. secretion. Cytokine Growth Factor Rev..

[65] Mousavijazi M., Naderan A., Ebrahimpoor M., Sadeghipoor M. (2013). Association between psychological stress and stimulation of inflammatory responses in periodontal disease. J. Dent..

[66] Kiecolt-Glaser J.K., Loving T.J., Stowell J.R., Malarkey W.B., Lemeshow S., Dickinson S.L., Glaser R. (2005). Hostile marital interactions, pro-inflammatory cytokine production, and wound healing. Arch. Gen. Psychiatry.

[67] Ferland C.L., Schrader L.A. (2011). Regulation of histone acetylation in the hippocampus of chronically stressed rats: A potential role of sirtuins. Neuroscience.

[68] Hunter R.G., Mc Carthy K.J., Milne T.A., Pfaff D.W., McEven B.S. (2009). Regulation of hippocampal H3 histone methylation by acute and chronic stress. Proc. Natl. Acad. Sci. USA.

[69] Genco R.J., Ho A.W., Kopman J., Grossi S.G., Dunford R.G., Tedesco L.A. (1998). Models to evaluate the role of stress in periodontal disease. Ann. Periodontol..

[70] Anttila S.S., Knuuttila M.L., Sakki T.K. (2001). Relationship of depressive symptoms to edentulousness, dental health, and dental health behavior. Acta Odontol. Scand..

[71] Costa F.O., Cota L.O.M. (2019). Cumulative smoking exposure and cessation associated with the recurrence of periodontitis in periodontal maintenance therapy: A 6-year follow-up. J. Periodontol..

[72] Copeland L.B., Krall E.A., Brown L.J., Garcia R.I., Streckfus C.F. (2004). Predictors of tooth loss in two US adult populations. J. Public Health Dent..

[73] Foster J.A., Rinaman L., Cryan J.F. (2017). Stress & the gut-brain axis: Regulation by the microbiome. Neurobiol. Stress.

[74] Christ A., Lauterbach M., Latz E. (2019). Western diet and the immune system: An inflammatory connection. J. Immun..

[75] Gur T.L., Worly B.L., Bailey M.T. (2015). Stress and the commensal microbiota: Importance in parturition and infant neurodevelopment. Front. Psychiatry.

[76] Barone A., Chatelain S., Derchi G., Di Spirito F., Martuscelli R., Porzio M., Sbordone L. (2020). Effectiveness of antibiotics in preventing alveolitis after erupted tooth extraction: A retrospective study. Oral Dis..

[77] Di Spirito F., Argentino S., Martuscelli R., Sbordone L. (2019). MRONJ incidence after multiple teeth extractions in patients taking oral bisphosphonates without “drug holiday”: A retrospective chart review. Oral Implantol..

[78] Lindhe J., Liljenberg B., Listgarten M. (1980). Some microbiological and histopathological features of periodontal disease in man. J. Periodontol..

[79] Rai B., Kaur J., Anand S.C., Jacobs R. (2011). Salivary stress markers, stress, and periodontitis: A pilot study. J. Periodontol..

[80] Yost S., Duran-Pinedo A.E., Teles R., Krishnan K., Frias-Lopez J. (2015). Functional signatures of oral dysbiosis during periodontitis progression revealed by microbial metatranscriptome analysis. Genome Med..

[81] Duran-Pinedo A.E., Chen T., Teles R., Starr J.R., Wang X., Krishnan K., Frias-Lopez J. (2014). Community-wide transcriptome of the oral microbiome in subjects with and without periodontitis. ISME J..

[82] Irwin M., Patterson T., Smith T.L. (1990). Reduction of immune function in life stress and depression. Biol. Psychiatry.

[83] Mesa F., Magán-Fernández A., Muñoz R. (2014). Catecholamine metabolites in urine, as chronic stress biomarkers, are associated with higher risk of chronic periodontitis in adults. J. Periodontol..

[84] Mazza M.G., De Lorenzo R., Conte C., Poletti S., Vai B., Bollettini I., Melloni E.M.T., Furlan R., Ciceri F., Rovere-Querini P. (2020). Anxiety and depression in COVID-19 survivors: Role of inflammatory and clinical predictors. Brain Behav. Immun..

[85] Cameron M.J., Bermejo-Martin J.F., Danesh A. (2008). Human immunopathogenesis of severe acute respiratory syndrome (SARS). Virus Res..

[86] Tian R., Hou G., Li D., Yuan T.-F. (2014). A Possible Change Process of Inflammatory Cytokines in the Prolonged Chronic Stress and Its Ultimate Implications for Health. Sci. World J..

[87] Amato M., Zingone F., Caggiano M., Iovino P., Bucc C., Ciacci C. (2017). Tooth Wear Is Frequent in Adult Patients with Celiac Disease. Nutrients.

[88] Sudhanshu A., Sharma U., Vadiraja H.S., Ran K.R., Singhal R. (2017). Impact of yoga on periodontal disease and stress management. Int. J. Yoga.

[89] Kloostra P.W., Eber R.M., Inglehart M.R. (2007). Anxiety, stress, depression and patients’ responses to periodontal treatment: Periodontists’ knowledge and professional behavior. J. Periodontol..

[90] Feeney S.L. (2004). The relationship between pain and negative affect in older adults: Anxiety as a predictor of pain. J. Anxiety Disord..

[91] Kiecolt-Glaser J.K., Marucha P.T., Malarkey W.B., Mercado A.M., Glaser R. (1995). Slowing of wound healing by psychological stress. Lancet.

[92] Takada T., Yoshinari N., Sugiishi S., Kawase H., Yamane T., Noguchi T. (2004). Effect of restraint stress on progression of experimental periodontitis in rats. J. Periodontol..

[93] Pisano M., Amato A., Sammartino P., Iandolo A., Martina S., Caggiano M. (2021). Laser Therapy in the Treatment of Peri-Implantitis: State-of-the-Art, Literature Review and Meta-Analysis. Appl. Sci..

[94] Werner H., Hakeberg M., Dahlström L., Eriksson M., Sjögren P., Strandell A., Svanberg T., Svensson L., Wide Boman U. (2016). Psychological Interventions for Poor Oral Health: A Systematic Review. J. Dent. Res..

[95] Vernon L.T., Howard A.R. (2015). Advancing Health Promotion in Dentistry: Articulating an Integrative Approach to Coaching Oral Health Behavior Change in the Dental Setting. Curr. Oral Health Rep..

[96] Newton J.T., Asimakopoulou K. (2015). Managing oral hygiene as a risk factor for periodontal disease: A systematic review of psychological approaches to behaviour change for improved plaque control in periodontal management. J. Clin. Periodontol..

[97] Schwarz F., Derks J., Monje A., Wang H.L. (2018). Peri-implantitis. J. Periodontol..

[98] Vaz P., Gallas M.M., Braga A.C., Sampaio-Fernandes J.C., Felino A., Tavares P. (2012). IL1 gene polymorphisms and unsuccessful dentalimplants. Clin. Oral Implant. Res..

[99] Sampaio Fernandes M., Vaz P., Braga A.C., Sampaio Fernandes J.C., Figueiral M.H. (2017). The role of IL-1 gene polymorphisms (IL1A, IL1B, and IL1RN) as a risk factor in unsuccessful implants retaining overdentures. J. Prosthodont. Res..

